# Phenotypic Clustering and Fibroin Gene Expression Divergence in Romanian and Imported *Bombyx mori* Breeds Under Standardized Rearing

**DOI:** 10.3390/insects17070665

**Published:** 2026-06-25

**Authors:** Gabriela-Maria Baci, Adrian Ionașcu, Attila Cristian Rațiu, Daniel Severus Dezmirean

**Affiliations:** 1 Faculty of Animal Sciences and Biotechnologies, University of Agricultural Sciences and Veterinary Medicine of Cluj-Napoca, 3-5 Calea Mănăștur, 400372 Cluj-Napoca, Romania; gabriela-maria.baci@usamvcluj.ro (G.-M.B.); ddezmirean@usamvcluj.ro (D.S.D.); 2 Department of Genetics, Faculty of Biology, University of Bucharest, 1-3 Intrarea Portocalelor, 060101 Bucharest, Romania; a.ionascu20@s.bio.unibuc.ro

**Keywords:** *phenotypic clustering*, economic traits, gene expression profiling, *Fib-l*, *FIBH*, silkworm

## Abstract

The economic value of *Bombyx mori* is built on two complementary substrates: the productive traits and the molecular mechanism of fibroin synthesis. Despite their importance to European sericulture, neither phenotypic nor transcriptional characterizations of Romanian breeds have been reported. Herein, we combine ten phenotypic traits with qRT-PCR quantification of *fibroin light-chain* (*Fib-l*) and *fibroin heavy-chain* (*FIBH*) expression across four *B. mori* breeds: the Romanian breeds Băneasa 1 (B1) and Galben de Băneasa (GB), the Japanese breed JH3, and the Chinese breed Auriu Chinez (ACH). All breeds were reared on the same artificial diet under identical thermohydrometric conditions, highlighting the genetic background as the dominant source of variation. Phenotypically, univariate testing and multivariate analyses converged on a two-cluster solution, (B1 + JH3) and (GB + ACH), consistent with genetic background and silk-pigment. However, B1 is the only breed that combines high silk-gland mass with *Fib-l* overexpression relative to the other three breeds (FC = 2.7–3.6, all *p* < 1 × 10^−4^) and with a *Fib-l*/*FIBH* expression ratio of 9.27, above the 1.5–2.9 observed in the other three breeds. The molecular signature identifies B1 as transcriptionally distinct and provides a candidate mechanism for superior productive parameters under artificial-diet rearing.

## Simple Summary

The silk produced by the silkworm, *Bombyx mori*, remains one of the key players in terms of natural fibers that exhibit not only a tremendous impact on the economy, but also in life sciences areas. Improving silk production depends on identifying the breeds that exhibit superior productive potential. Despite the importance of Romanian sericulture, up to this moment, no comprehensive data has been reported on Romanian silkworm breeds. In this study, we characterized two Romanian breeds and also Japanese and Chinese-originated breeds, reared under identical conditions. We assessed the economic parameters and the expression profile of the key genes involved in fibroin production. The results revealed that the Romanian breed Băneasa 1 (B1) stands out in terms of silk gland development, productive traits, and unique molecular profile. While the breeds can be grouped based on the color of the silk produced, breed B1 displayed a particularly valuable genetic resource. Herein, we highlight the importance of integrating assessment of both productive traits and molecular profiles to be exploited in future breeding programs.

## 1. Introduction

The domestic silkworm *Bombyx mori* is the only fully domesticated lepidopteran and the single insect species supporting an agricultural-industrial chain that yields raw silk and an expanding range of biomaterials with medical, pharmaceutical, and biotechnological applications [1,2,3,4,5,6,7,8,9,10,11,12,13]. The molecular substrate of this value is the silk fibroin assembly, whose architecture has been characterized in detail: a light chain (Fib-l) and a heavy chain (FIBH) are linked at the C-terminus by a single disulfide bond, with a P25 glycoprotein attached non-covalently, the three components assembling in a 6:6:1 molar ratio into a ~2.3 MDa elementary unit secreted by the posterior silk gland (PSG) during the fifth larval instar [1]. The *Fib-l* gene maps to chromosome 14 and *FIBH* to chromosome 25 [2,3]; both are massively transcribed in the PSG, and their relative expression has been proposed as a molecular marker for breed-specific silk properties [14].

The exploitation of *B. mori* critically depends on the availability of phenotypically and genetically distinct breeds whose productive and molecular characteristics have been evaluated under standardized conditions [15,16,17]. Although over time, artificial selection has improved the productive performance for more than 1000 *B. mori* breeds maintained worldwide, it has also reduced genetic diversity [18]. As a result, a wide range of breeds reared under comparable conditions tend to stabilize for terminal cocoon parameters [15], while early developmental and silk-gland traits retain measurable inter-breed variation that breeding programs can exploit [16,17]. Quantifying both the phenotypic and the transcriptional layers in the same experimental framework is therefore an essential step for any informed selection strategy.

Two of the most important Romanian breeds, namely Băneasa 1 (B1) and Galben de Băneasa (GB), are kept at the Global Centre of Excellence for Advanced Research in Sericulture and Promotion of Silk Production (GCEARS-PSP, University of Agricultural Sciences and Veterinary Medicine of Cluj-Napoca). These local stocks are routinely paired with imported lineages—among which the Japanese breed JH3 and the Chinese breed Auriu Chinez (ACH)—for hybridization and crossbreeding. A previous molecular characterization of Romanian breeding and hybrid lines by our group outlined the genetic background for B1 and grouped GB with naturally yellow-cocoon lineages [19], but no quantitative side-by-side characterization of either the productive traits or the fibroin transcript profiles of these four breeds has been published when they are reared under a single uniform regime.

Phenotypic expression in *B. mori* is strongly modulated by environment, temperature, humidity, and nutrition [20,21,22], and especially by artificial versus mulberry-leaf rearing [23,24,25,26], with environmental contributions often outweighing genetic background for economic traits such as cocoon weight and shell ratio [22]. We therefore performed an experiment in which the four breeds were reared simultaneously, on the same artificial diet, in the same season and under identical thermohydrometric conditions, so that residual phenotypic and transcriptional variation could be attributed primarily to genetic background. The aim of the present study is threefold: (i) to provide the first parallel single-regime quantitative characterization of B1, GB, JH3 and ACH for ten developmentally and economically relevant traits; (ii) to determine, by combining univariate testing with multivariate ordination and supervised classification, which traits separate the breeds and which are phenotypically canalized; and (iii) to test whether the phenotypic clustering is mirrored or contradicted at the transcriptional level by the expression profile of the two fibroin genes *Fib-l* and *FIBH* in the posterior silk gland.

## 2. Materials and Methods

### 2.1. Biological Material

**Figure 1:**
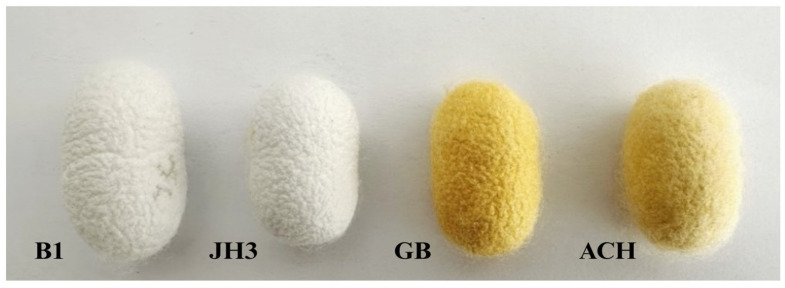
Phenotypic overview of the four *B. mori* breeds.

Four *B. mori* breeds were evaluated in parallel: the Romanian breeds B1 and GB, the Japanese breed JH3 and the Chinese breed ACH. All four are maintained as pure lines at GCEARS-PSP and are summarized in Table 1; cocoon-color and genetic-background information are derived from the molecular characterization of Romanian breeding lines by Furdui et al. [19]. Representative cocoons of the four breeds, photographed at the end of the spinning period, are shown in Figure 1; the white cocoon color of B1 and JH3 contrasts visually with the natural yellow pigmentation of GB and ACH, a trait whose biochemical determinants in domestic silk have been characterized in [27].

### 2.2. Egg Incubation and Larval Rearing

Disease-free *B. mori* eggs of each of the four breeds were obtained from GCEARS-PSP (International Sericultural Commission structure). Eggs were incubated under hygienic conditions at 24 ± 1 °C and 75–80% relative humidity. The rearing experiment was conducted in November, with all four breeds reared simultaneously in the same facility on an artificial diet supplied by the Agricultural Academy Scientific Centre on Sericulture (Vratsa, Bulgaria). First-instar larvae were reared at 28 °C and 80–90% humidity; 1 °C was decreased with each successive instar, and humidity was reduced to 70–80% during the fourth and fifth instars. The imported breeds (JH3, ACH) were reared under the same Romanian climatic conditions as the local breeds, removing rearing-environment effects from the inter-breed comparison.

### 2.3. Assessment of Economic Traits

Ten biological and economically relevant traits were recorded across the developmental cycle: incubation period (days; egg stage), larval instar duration (days), larval length (mm), larval weight (g), silk-gland weight (g; measured by dissection on day 5 of the fifth instar), cocoon transverse axis (mm), cocoon longitudinal axis (mm), cocoon weight (g) and pupal weight (g) after the completion of spinning, and female fecundity (total number of laid eggs per female) after adult emergence. To accommodate stage-specific destructive sampling, the 20 measurements per breed for each trait were obtained from independent randomly selected individuals (*n* = 20 per trait per breed). Raw measurements are provided in Supplementary Table S1.

### 2.4. qRT-PCR Analysis of Fib-l and FIBH

#### 2.4.1. Tissue Sampling and RNA Extraction

Posterior silk glands were dissected on the fifth day of the fifth instar, the developmental stage at which fibroin secretion attains its maximal level [28], and immediately preserved in RNA Save (Sartorius, Beit Haemek, Israel). Across all four breeds, the fifth-instar window represents 42–50% of the breed-specific instar span, a difference small enough that no breed-specific tuning of the sampling age was applied. For each breed, three biological replicates were processed, each pool consisting of posterior silk glands harvested from three larvae. Total RNA was extracted with the RNeasy Mini Kit (Qiagen, Hilden, Germany). following the manufacturer’s protocol; RNA concentration and purity were determined on a NanoVue spectrophotometer (BioChrom, Cambridge, United Kingdom). cDNA was reverse transcribed from 500 ng of total RNA using the SuperScript II Reverse Transcriptase kit (Invitrogen, Thermo Fisher Scientific, Carlsbad, CA, USA) with random primers (Promega, Madison, WI, USA).

#### 2.4.2. Primer Design

Primers for *Fib-l* and *FIBH* were designed against the *B. mori* reference assembly Bmori_2016v1.0 (RefSeq GCF_014905235.1), specifically chromosome 14 (NC_051371.1, Fib-l locus) and chromosome 25 (NC_051382.1, FIBH locus). The *Fib-l* primers bind to adjacent exons (exons 3 and 4) and yield a 169 bp amplicon, whereas the *FIBH* primers bind within the second exon of *FIBH* and produce a 141 bp amplicon. Both amplicon lengths were matched to the 147 bp amplicon of the previously validated *Actin A3* endogenous reference gene primers (F: 5′-CGGCTACTCGTTCACTACC-3′; R: 5′-CCGTCGGGAAGTTCGTAAG-3′; [29]). Primer details are summarized in Table 2.

#### 2.4.3. qRT-PCR Amplification

Three technical replicates were run for each gene (*Fib-l*, *FIBH*, *Actin A3*) per biological replicate, yielding nine Ct values per gene per breed. The 20 μL reaction mix contained 2 μL (20 ng) cDNA template, 0.32 μL of each forward and reverse primer (Generi Biotech, Hradec Králové, Czech Republic), 10 μL of 1× GoTaq qRT-PCR Master Mix (Promega), and 7.36 μL PCR-grade H_2_O. Amplification was performed on a 7500 Real-Time PCR System (Applied Biosystems) under the following program: 95 °C for 3 min; 40 cycles of 95 °C for 23 s, 59 °C for 30 s, 72 °C for 32 s (data acquisition); final stage 95 °C for 15 s followed by 60 °C for 1 min.

#### 2.4.4. Expression Analysis

Inter-breed fold-change (FC) values were computed by the 2^−ΔΔCt^ method [30] in the qDATA bioinformatics tool [31], using *Actin A3* as the endogenous reference [29]. Statistical significance of expression differences between breed pairs was assessed by Welch’s *t*-tests on the 27 individual 2^−ΔΔCt^ values per condition (three biological × three technical × three Ct readings) [31]. Mean ± SE FC values were visualized with qDATA and GraphPad Prism 8.4.2 (GraphPad Software, Boston, MA, USA).

### 2.5. Statistical Analysis

All phenotypic analyses were performed in R 4.3.1 [32] within RStudio 2023.06.2 [33], using dplyr [34], ggplot2 [35], nortest [36], tseries [37], car [38], stats [32], FactoMineR [39], factoextra [40], and KODAMA [41,42]. Univariate normality was assessed by Shapiro–Wilk and Kolmogorov–Smirnov tests; variance homogeneity by Levene’s test. Inter-breed comparisons for each phenotypic trait were performed in parallel by one-way ANOVA, Welch’s ANOVA (robust to heteroscedasticity), and Kruskal–Wallis (non-parametric), followed by Tukey’s HSD pairwise post hoc. Multivariate structure was investigated by Principal Component Analysis (PCA) on the full individual dataset and on breed means, by Ward hierarchical clustering on standardized breed means, and by the KODAMA-t-SNE algorithm [41,42]. Supervised classification was tested by Linear Discriminant Analysis (LDA) and Random Forest, both evaluated by leave-one-out cross-validation. Multivariate phenotypic analyses (PCA, Ward hierarchical clustering, KODAMA-t-SNE, LDA, and Random Forest with leave-one-out cross-validation) were performed with the assistance of Claude (Anthropic, Inc.; models claude-opus-4-6 and claude-sonnet-4-6). Statistical significance was set at *p* < 0.05.

## 3. Results

### 3.1. Univariate Trait Comparison Among Breeds

Mean values and standard errors for the ten measured traits are summarized in Table 3. The four breeds spanned a clear gradient for egg-stage and larval-stage traits: B1 had the shortest incubation period (9.95 ± 0.16 days) and the heaviest fifth-instar larvae (3.02 ± 0.103 g), while ACH showed the opposite extremes (13.06 ± 0.10 days incubation; 2.25 ± 0.02 g larval weight). Silk-gland mass followed the same ordering (B1 > JH3 > GB > ACH) (Supplementary Figures S1 and S2).

Because several traits, including the count-based variables (incubation period, larval duration) and cocoon weight, deviated from normality in at least one breed (Shapiro–Wilk *p* < 0.05), all univariate inter-breed comparisons were performed in parallel by the one-way ANOVA, Welch’s ANOVA (which does not assume variance homogeneity), and the Kruskal–Wallis non-parametric test (Table 4).

All seven traits with a developmental or productive component yielded concordant, significant results across the three tests. Among the architectural cocoon traits, the cocoon transverse axis was significant only under Kruskal–Wallis, and pupal weight and cocoon weight failed to reach significance under any test, indicating that the four breeds are statistically indistinguishable for these terminal mass and architectural variables.

Pairwise Tukey HSD comparisons (Table 5) localized the inter-breed differences. The B1-JH3 pair and the GB-ACH pair showed few significant differences between member breeds, but each of the (B1, JH3) pair differed highly significantly from each of the (GB, ACH) pair on the larval-development axis. Cocoon transverse axis showed no significant pairwise difference (all *p* > 0.05). Fecundity displayed a non-pairwise pattern: GB was significantly lower than each of the other three breeds, none of which differed from each other.

### 3.2. Multivariate Structure of the Four-Breed Phenotypic Variation

**Figure 2:**
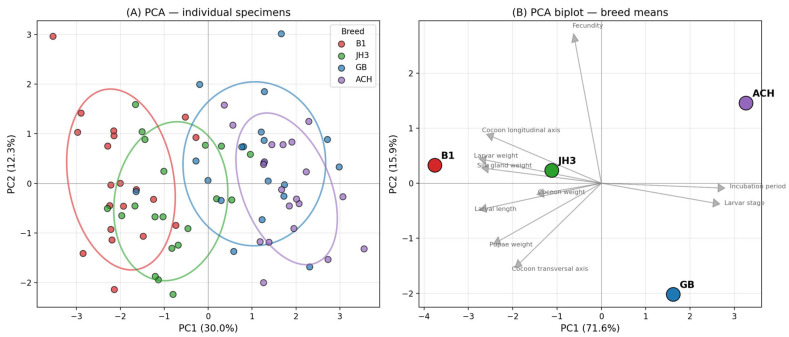
Principal Component Analysis. (**A**) PCA on the full individual dataset (*n* = 80, with 1.5 σ confidence ellipses); PC1 = 29.5%, PC2 = 12.3%. (**B**) PCA biplot on the four breeds means: PC1 = 71.6%, PC2 = 15.9% (87.6% cumulatively).

PCA on the standardized individual dataset (80 individuals × 10 traits) yielded PC1 = 29.5% and PC2 = 12.3% of total variance (Figure 2A), with the breeds separated along PC1 into three visually identifiable clouds: B1, JH3, and a partially overlapping (GB, ACH) group. The five traits that contributed most to PC1—incubation period, larval weight, larval length, larval duration, and silk-gland weight—are precisely the same parameters yielding concordant significance across all three univariate tests, while the three non-significant terminal architectural traits (cocoon transverse axis, cocoon weight, pupal weight) ranked lowest in their PC1 contributions, providing a self-consistent validation of the univariate findings.

On the four-breed means (4 × 10 matrix), PC1 captured 71.6% and PC2 15.9% of the inter-breed variance (Figure 2B). Because breed means reducing the dataset to four observations, the effective rank of the standardized matrix is at most three, so the large proportion of variance explained by PC1 reflects in part an algebraic constraint rather than solely a biological signal; the biplot is therefore presented as a visualization of inter-breed trait-profile distances rather than an inferential result. B1 and JH3 clustered on the negative side of PC1, driven by their high larval mass, silk-gland weight, and larval length, the same traits that separate these two breeds from the others in the univariate analysis. GB and ACH occupied the positive side of PC1, reflecting their longer incubation period and larval development duration; JH3 held an intermediate position. Along PC2, Fecundity was the dominant contributor: ACH, the highest egg-layer in the panel (401.1 ± 15.4 eggs/female), scored on positive PC2, while GB, with the lowest fecundity (318.2 ± 10.2 eggs/female), scored on negative PC2, a pairwise difference confirmed as significant by Tukey HSD (*p* < 0.001; Table 5).

**Figure 3:**
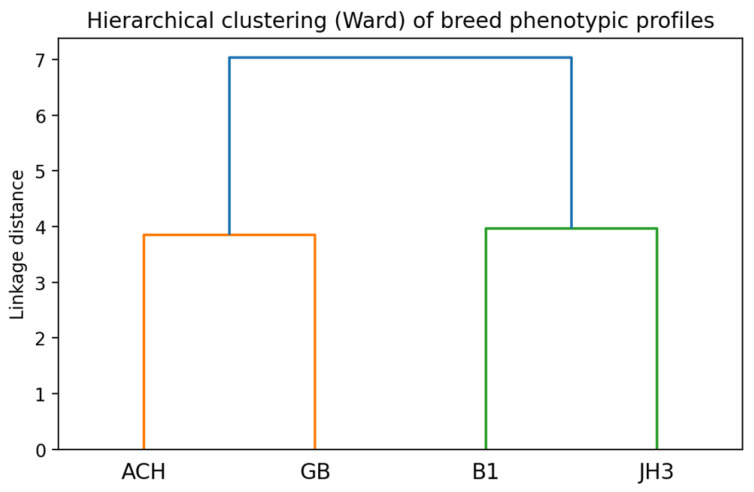
Hierarchical clustering (Ward linkage) of the four breed phenotypic profiles on the matrix of breed means after standardization.

Hierarchical clustering by Ward linkage on the standardized breed mean profiles produced a dendrogram (Figure 3) fully concordant with the PCA ordination. Two clusters formed: ACH and GB merged at a linkage distance of approximately 3.9, while B1 and JH3 merged at a near-identical distance of approximately 4.0. The two clusters themselves fused at a linkage distance of approximately 7, roughly twice the within-cluster distances, indicating that the separation between the (ACH, GB) and (B1, JH3) groups is substantially larger than the internal cohesion of either pair. The near-symmetry of the two within-cluster distances further suggests that the degree of phenotypic similarity is comparable within each pair, consistent with their shared genetic backgrounds.

**Figure 4:**
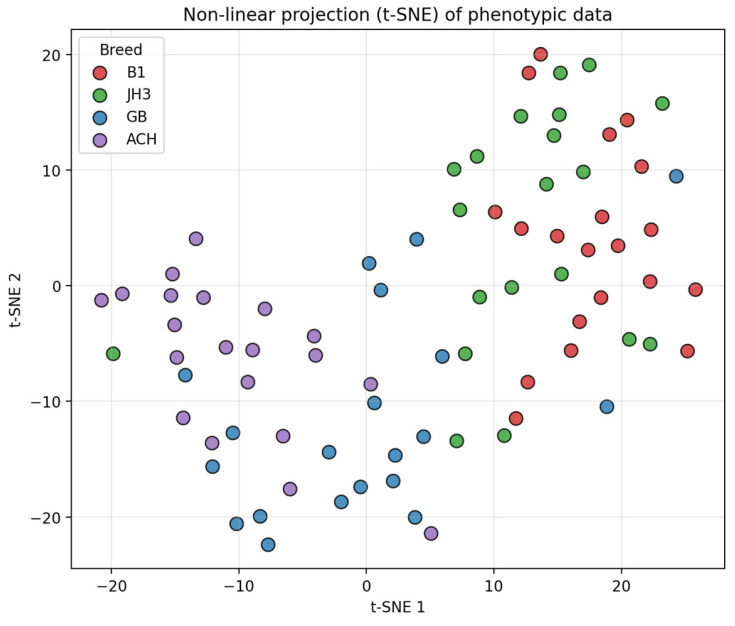
KODAMA-t-SNE projection of the individual dataset (perplexity = 10).

To confirm the two-cluster structure at the level of individual specimens rather than breed means, a KODAMA-t-SNE projection was applied to the full individual dataset (Figure 4; perplexity = 10). The resulting plot placed B1 and JH3 individuals predominantly in the right portion of the embedding space (positive t-SNE 1), while GB and ACH individuals occupied the left portion (negative t-SNE 1), recapitulating the two-cluster partition identified by PCA and Ward clustering. Substantial within-cluster scatter and partial inter-breed overlap are visible for both pairs, which is expected given the intra-breed individual variability documented in the univariate distributions (Table 3) and reflects the biological reality of a continuous, quantitative phenotypic space rather than discrete breed-level discontinuities.

Internal cluster validation by k-means silhouette analysis on the standardized dataset further supported the two-cluster solution (silhouette = 0.21 for k = 2; 0.16 for k = 3; 0.13 for k = 4). Leave-one-out cross-validation (LOOCV) of a Linear Discriminant Analysis correctly assigned 61 of 80 individuals to their breed of origin (76.2% accuracy), and an independent LOOCV Random Forest classifier reached 65%; both values vastly exceed the 25% chance level for a four-class problem.

### 3.3. Fibroin Gene Expression Profile in the Posterior Silk Gland

**Figure 5:**
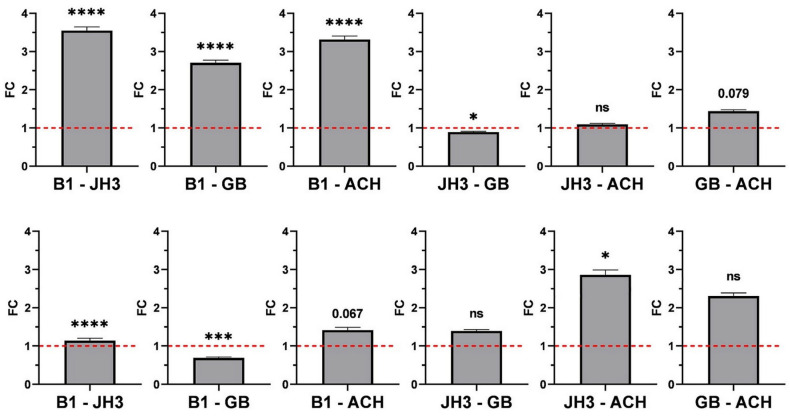
Pairwise fold-change (mean ± SE) of *Fib-l* and *FIBH* expression between *B. mori* breeds. The red dashed line marks the FC = 1 baseline, determining if the comparisons show up-regulation (FC > 1) or down-regulation (FC < 1). Statistical significance: ns ≥ 0.05, * < 0.05, *** < 0.001 and **** < 0.0001.

**Figure 6:**
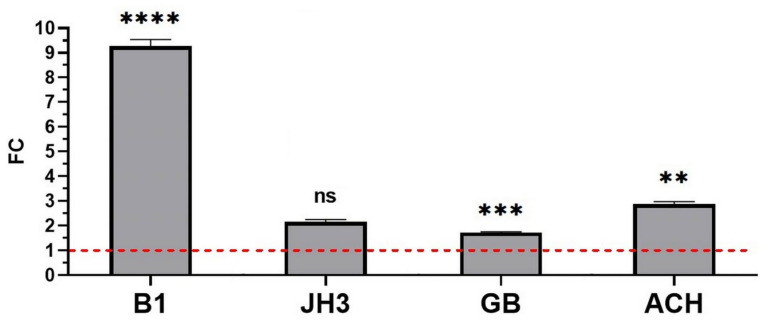
Within-breed *Fib-l*/*FIBH* expression ratio (FC). B1 expresses *Fib-l* mRNA at 9.27-fold the level of *FIBH* mRNA, far above the 1.7–2.9 ratios measured in JH3, GB, and ACH. Statistical significance: ns ≥ 0.05, ** < 0.01, *** < 0.001 and **** < 0.0001. Error bars are shown as SE. The dotted red line marks the up/down-regulation threshold for calculated FC values.

To probe whether the phenotypic clustering described in Section 3.1 and Section 3.2 is mirrored at the transcriptional level, we quantified the steady-state expression of the two fibroin genes *Fib-l* and *FIBH* in the posterior silk gland on day 5 of the fifth instar, the developmental window of maximal fibroin secretion [28], across the four breeds. Pairwise fold-change (FC) values for the six breed comparisons are reported in Table 6 and Figure 5; the relative *Fib-l-*vs-*FIBH* expression within each breed is shown in Figure 6.

The most striking pattern is the systematic and highly significant overexpression of *Fib-l* in B1 relative to every other breed (FC = 2.706 vs. GB, *p* < 10^−4^; FC = 3.317 vs. ACH, *p* < 10^−5^; FC = 3.552 vs. JH3, *p* < 10^−5^). By contrast, JH3, GB, and ACH cluster within a narrow range of *Fib-l* expression, with only the JH3 vs. GB comparison reaching nominal significance (FC = 0.895, *p* = 0.019) and the GB vs. ACH comparison being borderline (FC = 1.443, *p* = 0.079). For *FIBH*, the picture inverts: B1 was modestly up-regulated relative to JH3 (FC = 1.141, *p* < 10^−3^) and modestly down-regulated relative to GB (FC = 0.688, *p* ≈ 10^−3^), with the largest *FIBH* effect being the JH3 vs. ACH overexpression (FC = 2.862, *p* = 0.04). The two genes are thus regulated asymmetrically: B1 is a clear *Fib-l*-high outlier, whereas GB is the highest expressor of *FIBH*.

The within-breed comparison of *Fib-l* vs. *FIBH* expression (Figure 6) sharpens this picture decisively: B1 displays a *Fib-l*/*FIBH* mRNA ratio of 9.27 (*p* < 10^−6^), more than three-fold higher than the next-ranked breed (ACH, 2.87, *p* < 10^−2^; JH3, 2.15, n.s.; GB, 1.71, *p* < 10^−3^). B1 is therefore the only breed in the panel whose silk-gland transcript pool is dominated to such an extent by the light fibroin chain, a configuration that departs markedly from the 6:6:1 Fib-l:FIBH:P25 protein stoichiometry classically reported for the assembled fibroin elementary unit [4].

## 4. Discussion

The key finding that emerges from a single-regime parallel rearing of the four breeds is that the genetic background of *B. mori* remains a strong determinant of early developmental and silk-gland phenotypes, even after millennia of artificial selection have homogenized cocoon mass and architecture. Of the ten phenotypic traits measured, seven (all the developmental and productive variables) yielded *p* < 10^−3^ across all three univariate tests, while the three terminal architectural variables (cocoon transverse axis, cocoon weight, pupal weight) failed to discriminate the breeds. This dichotomy mirrors the two-tier model documented in the comparative literature: Zanatta et al. [15] reported considerable inter-breed variability for larval body weight and raw silk parameters across 16 parental strains, and Bhat et al. [16] identified strong variability for fecundity and hatching across 18 breeds, but both studies documented far smaller breed effects on cocoon mass than on larval traits. Zamani et al. [17] further showed that even narrowly defined selection programs targeting cocoon-shell weight required generations of directional pressure to overcome the apparent canalization of this trait.

Two non-mutually exclusive mechanisms plausibly underlie the conservation of cocoon architecture across the four breeds. First, the figure-eight spinning motion that builds the *B. mori* cocoon imposes a biomechanical envelope on transverse dimensions, which are mechanically coupled to larval body size and to the liquid-crystalline rheology of fibroin extrusion [42,43,44]. Second, intensive selection over the domestication history of *B. mori* has progressively narrowed the genetic diversity of all domestic stocks and converged terminal-trait distributions on a productive optimum [18]; pupae that are too heavy or cocoons whose walls are too thick experience impaired gas exchange and reduced viability, imposing a stabilizing upper bound. Importantly, environmental rather than genetic factors dominate the residual variance of cocoon weight and shell ratio under typical sericultural conditions [22]. In our experiment, all four breeds were reared on the same artificial diet, in the same facility, in the same season, and under identical thermohygrometric ramps; under such uniform conditions, the genetic signal alone is insufficient to displace these traits from their canalized optima, which is precisely what we observe. Artificial diet itself further compresses the dynamic range of productive traits [23,24], and supplementation strategies tested on the GCEARS-PSP stocks have shown that bee-pollen enrichment fails to recover this loss [26], whereas zinc supplementation specifically modifies silk-gland and cocoon mass [25].

The phenotypic multivariate analyses provide a coherent biological interpretation of the breed groupings. The (GB, ACH) cluster shares the synthesis of natural yellow silk pigment, a trait whose biochemical determinants have been characterized in domestic silk by Ma et al. [27] and which reflects shared genomic architecture; the consistent grouping across PCA, Ward clustering, KODAMA-t-SNE and Tukey HSD therefore reflects an ancestral commonality rather than an artifact of analytical choice. The (B1, JH3) cluster, in turn, is concordant with the molecular characterization of Furdui et al. [19], who documented a Japanese genetic background for B1 and grouped it with other Japanese-origin Romanian lines. The quantitative output of the Ward dendrogram reinforces this interpretation: the within-cluster linkage distances for (ACH, GB) and (B1, JH3) are nearly equal (≈3.9 and ≈4.0, respectively), indicating comparable internal phenotypic cohesion within each pair, while the inter-cluster distance (≈7.0) is roughly 1.75-fold larger, providing a metric confirmation that the separation between the two groups substantially exceeds their internal heterogeneity. The symmetry of within-cluster distances is notable: despite differing geographic origins and breeding histories, neither breed pair is detectably more internally uniform than the other, suggesting that the domestication-driven phenotypic convergence has operated similarly within each lineage. At the individual-specimen level, the KODAMA-t-SNE projection (Figure 4) confirms the two-cluster partition while also exposing the within-breed scatter that underlies the 76% LOOCV classification accuracy rather than a higher value: B1 and JH3 individuals overlap partially within their shared region of the embedding space, as do GB and ACH, a pattern fully consistent with the intra-breed phenotypic distributions documented in Table 3 and with the known biological reality that quantitative trait variation within a breed is continuous rather than discrete. The fact that four structurally independent analytical approaches—Tukey HSD pairwise testing, PCA ordination, Ward hierarchical clustering, and KODAMA-t-SNE non-linear projection—converge on the same two-group solution is strong evidence that the cluster signal is a genuine property of the data rather than a methodological artifact of any particular algorithm.

The most informative finding of the present study, however, emerges from the integration of the phenotypic and the transcriptional layers. Phenotypically, B1 and JH3 cluster together (Figure 2, Figure 3 and Figure 4) and share similar silk-gland masses (0.83 vs. 0.81 g). At the transcript level, however, B1 separates sharply from JH3 and from every other breed through a 2.7–3.6-fold overexpression of the *Fib-l* gene (all *p* < 10^−4^; Figure 5) and through a *Fib-l*/*FIBH* expression ratio of 9.27, more than threefold above the next-ranked breed (Figure 6). This is a clear example of phenotype-to-transcript dissociation: the molecular signature of B1 is detectable in the silk gland even when its macroscopic productive traits would group it with JH3. Conversely, the *FIBH* gene displays a different breed ordering, with GB as the highest expressor, consistent with the asymmetric regulation of the two fibroin chains and with Naik et al.’s proposal that *Fib-l* and *FIBH* expression can serve as molecular markers for breed-specific silk properties [14].

**Figure 7:**
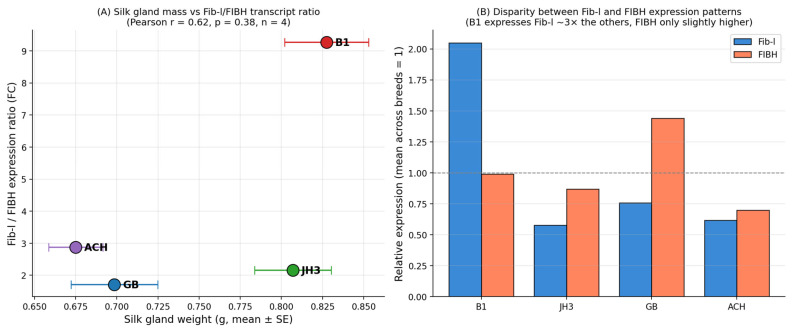
Phenotype-to-transcript integration. (**A**) Silk-gland mass (mean ± SE, *n* = 20) plotted against the within-breed *Fib-l*/*FIBH* expression ratio (Figure 6). The trend across the four breeds is positive (Pearson r = 0.62), with B1 as a marked outlier on both axes. (**B**) Relative expression of *Fib-l* and *FIBH* across breeds, rescaled to a between-breed mean of 1 for each gene; the two genes follow markedly different breed-level patterns.

It is worth contrasting our results with those of Fang et al. [45], who reported broadly similar expression levels of *Fib-l* and *FIBH* across the breeds they analyzed. In our panel, this 1:1 transcript balance is approached only by JH3 (*Fib-l*/*FIBH* ratio = 2.15); the other three breeds deviate from this balance to varying degrees, and B1 deviates massively. The canonical 6:6:1 Fib-l:FIBH:P25 stoichiometry of the assembled fibroin elementary unit [4] corresponds, at the protein level, to a 1:1 Fib-l:FIBH ratio; the transcript imbalance we observe in B1 therefore points either to a post-transcriptional buffering mechanism that restores the protein stoichiometry (most plausibly via differential translational efficiency or differential mRNA stability of the heavy chain) or to a genuinely modified protein composition of the B1 silk thread both hypotheses that warrant focused biochemical follow-up. Notably, the *Fib-l*/*FIBH* ratio across the four breeds correlates positively with silk-gland mass (Pearson r = 0.62, Figure 7A), suggesting that the elevated *Fib-l* dosage in B1 is biologically coupled to its larger PSG.

From a methodological standpoint, the consistency between univariate testing (which identifies the same five top traits as significant under parametric and non-parametric inference), multivariate ordination (which uses these same five traits to build >90% of PC1 in the means-PCA), supervised classification (76% LOOCV accuracy) and the qRT-PCR molecular layer (which singles out B1 even within the phenotype-similar (B1, JH3) cluster) provides mutually reinforcing evidence that the breed signal is robust and not an artifact of any single statistical choice. The increase in variance explained by PC1 from 29.5% (individuals) to 71.6% (breed means) is the expected mathematical consequence of averaging out intra-breed individual noise rather than an inflation of the result.

Two limitations should be acknowledged. First, the sample size of *n* = 20 per breed per phenotypic trait and three biological replicates per breed for qRT-PCR are sufficient for the present comparative aims, but at the lower bound of what would be required to characterize the full distribution of rarer phenotypes. Second, the inter-breed comparison is informative for the rearing conditions tested (artificial diet, November cycle, Romanian climate); breed × environment interaction terms could not be estimated here and represent a natural next step.

## 5. Conclusions

By combining a single-regime parallel rearing of two Romanian (B1, GB) and two reference imported (JH3, ACH) *B. mori* breeds with qRT-PCR quantification of the *Fib-l* and *FIBH* genes in the posterior silk gland, this study provides the first phenotype-to-transcript characterization of the GCEARS-PSP stock. The four breeds resolve into two phenotypic clusters (B1, JH3) and (GB, ACH), which are coherent with their documented genetic backgrounds and silk-pigment biochemistry [19,27] and that support a 76% LOOCV individual-to-breed classification. The transcriptional layer disrupts this symmetry: B1 alone combines high silk-gland mass with a massive *Fib-l* overexpression (FC 2.7–3.6 vs. each of the other breeds, all *p* < 10^−4^) and with a *Fib-l*/*FIBH* mRNA ratio of 9.27, well above the 1.5–2.9 of the other three breeds and above the 1:1 ratio implied by the classical 6:6:1 Fib-l:FIBH:P25 protein stoichiometry [4]. This molecular signature provides a candidate transcriptional substrate for B1’s superior productive performance under artificial-diet rearing and identifies the *Fib-l*/*FIBH* ratio as a useful breed-discriminating molecular marker, complementing the use of these genes proposed by Naik et al. [14]. The dataset establishes a quantitative phenotype-to-transcript baseline against which future hybridization, mulberry-feeding, and supplementation experiments on the Romanian breeding stock can be benchmarked.

## Figures and Tables

**Table 1:** Origin and qualitative descriptors of the four *B. mori* breeds compared in this study.

Code	Full Name	Geographic Origin	Cocoon Color
B1	Băneasa 1	Romania	White
GB	Galben de Băneasa	Romania	Yellow
JH3	JH3	Japan	White
ACH	Auriu Chinez	China	Yellow

**Table 2:** *Fib-l* and *FIBH* qRT-PCR primer sequences and their genomic coordinates on the *B. mori* reference assembly Bmori_2016v1.0 (RefSeq GCF_014905235.1).

Primer	Sequence (5′-3′)	Genomic Coordinates	Strand	Chromosome (RefSeq)
qRT-PCR_F_Fib-l	CGGAGTTGACCAGGGTTGAT	9,688,263–9,688,282	plus	14 (NC_051371.1)
qRT-PCR_R_Fib-l	GTATCCCCGGTGATGCCTGTG	9,689,083–9,689,065	minus	14 (NC_051371.1)
qRT-PCR_F_FIBH	ATGGACTCGTTACCGTCGGA	10,370,345–10,370,364	plus	25 (NC_051382.1)
qRT-PCR_R_FIBH	TGCATCTGGGGCAGTTATCG	10,370,485–10,370,466	minus	25 (NC_051382.1)

**Table 3:** Mean ± standard error for the ten measured phenotypic traits across the four B. mori breeds (*n* = 20 per breed per trait; missing values excluded). Bolded values mark the highest mean per trait.

Trait	B1	JH3	GB	ACH
Incubation period (days)	9.95 ± 0.16	10.88 ± 0.17	12.40 ± 0.15	13.06 ± 0.10
Larval weight (g)	3.02 ± 0.103	2.72 ± 0.096	2.31 ± 0.092	2.25 ± 0.086
Larval duration (days)	32.74 ± 0.24	32.89 ± 0.31	33.95 ± 0.14	34.11 ± 0.19
Silk gland weight (g)	0.83 ± 0.025	0.81 ± 0.023	0.70 ± 0.026	0.68 ± 0.017
Larval length (mm)	51.45 ± 0.20	50.38 ± 0.25	49.80 ± 0.29	48.76 ± 0.37
Cocoon transverse axis (mm)	15.20 ± 0.16	15.37 ± 0.15	15.18 ± 0.17	14.59 ± 0.26
Fecundity (eggs/female)	390.5 ± 11.7	387.0 ± 15.5	318.1 ± 10.2	401.1 ± 15.4
Pupal weight (g)	0.389 ± 0.021	0.352 ± 0.018	0.361 ± 0.023	0.325 ± 0.016
Cocoon longitudinal axis (mm)	32.23 ± 0.32	31.11 ± 0.38	30.26 ± 0.76	30.52 ± 0.36
Cocoon weight (g)	0.482 ± 0.024	0.412 ± 0.019	0.445 ± 0.028	0.436 ± 0.016

**Table 4:** One-way ANOVA, Welch’s ANOVA and Kruskal–Wallis (K-W) test of the four-breed comparison for each phenotypic trait. Levene’s test on cocoon longitudinal axis (*p* = 7 × 10^−4^) indicated heteroscedasticity, so Welch’s ANOVA result is the primary inference for that trait.

Trait	ANOVA F	*p*	Welch F	*p*	K-W χ^2^	*p*
Incubation period	91.47	<1 × 10^−16^	105.31	<1 × 10^−16^	58.66	1.1 × 10^−12^
Larval weight	14.78	1.1 × 10^−7^	13.86	2.0 × 10^−6^	31.12	8.0 × 10^−7^
Larval duration	10.02	1.3 × 10^−5^	10.00	5.5 × 10^−5^	25.08	1.5 × 10^−5^
Silk gland weight	10.82	5.4 × 10^−6^	12.24	7.2 × 10^−6^	25.95	9.8 × 10^−6^
Larval length	15.48	6.0 × 10^−8^	16.20	4.1 × 10^−7^	28.66	2.6 × 10^−6^
Cocoon transverse axis	3.20	0.028	2.25	0.097	12.51	5.8 × 10^−3^
Fecundity	7.98	1.1 × 10^−4^	10.80	2.2 × 10^−5^	20.00	1.7 × 10^−4^
Pupal weight	1.77	0.161	1.98	0.132	4.65	0.199
Cocoon longitudinal axis	3.18	0.029	4.89	5.3 × 10^−3^	10.77	0.013
Cocoon weight	1.63	0.189	1.64	0.194	4.99	0.173

**Table 5:** Tukey HSD *p*-values for all pairwise breed comparisons. Bold values indicate *p* < 0.05.

Trait	B1 vs. JH3	B1 vs. GB	B1 vs. ACH	JH3 vs. GB	JH3 vs. ACH	GB vs. ACH
Incubation period	0.0002	<1 × 10^−7^	<1 × 10^−7^	<1 × 10^−7^	<1 × 10^−7^	0.012
Larval weight	0.130	<1 × 10^−7^	<1 × 10^−7^	0.073	0.004	0.979
Larval duration	0.964	0.001	3 × 10^−4^	0.007	0.002	0.959
Silk gland weight	0.924	0.001	1 × 10^−4^	0.026	8 × 10^−4^	0.891
Larval length	0.045	6 × 10^−4^	<1 × 10^−7^	0.491	0.001	0.056
Cocoon transverse axis	0.915	0.999	0.119	0.896	0.025	0.133
Fecundity	0.998	0.001	0.944	0.003	0.879	2 × 10^−4^
Pupal weight	0.555	0.751	0.112	0.989	0.772	0.577
Cocoon longitudinal axis	0.377	0.029	0.074	0.614	0.830	0.982
Cocoon weight	0.139	0.655	0.479	0.740	0.883	0.992

**Table 6:** Pairwise fold-change (FC ± SE) for *Fib-l* and *FIBH* expression in the posterior silk gland of the four *B. mori* breeds at day 5 of the fifth instar. Statistical significance from Welch’s *t*-tests on 2^−ΔΔCt^ values (df = 52).

Comparison	*Fib-l* FC ± SE	*Fib-l p*	*FIBH* FC ± SE	*FIBH p*
B1 vs. JH3	3.552 ± 0.094	<1 × 10^−5^	1.141 ± 0.061	<1 × 10^−3^
B1 vs. GB	2.706 ± 0.070	<1 × 10^−4^	0.688 ± 0.023	≈1 × 10^−3^
B1 vs. ACH	3.317 ± 0.087	<1 × 10^−5^	1.417 ± 0.072	0.067
JH3 vs. GB	0.895 ± 0.019	0.019	1.390 ± 0.038	n.s.
JH3 vs. ACH	1.096 ± 0.023	0.515	2.862 ± 0.128	0.040
GB vs. ACH	1.443 ± 0.032	0.079	2.305 ± 0.084	n.s.

## Data Availability

Raw data are provided as Supplementary Table S1.

## Supplementary Materials

The following supporting information can be downloaded at: https://www.mdpi.com/article/10.3390/insects17070665/s1, Figures S1 and S2: Quantile-quantile (Q-Q) plots of each measured biological parameter corresponding to the four local *B. mori* breeds of interest: A-B1, B-JH3, C-GB, D-ACH. Each graph displays the linear relation between quantiles drawn from the parameter distribution and the normal distribution. All graphs were generated using the qqPlot() function from the EnvStats R package in RStudio; Table S1: Raw data underlying the experimental analysis.
