# Supplementary material for: Phenotypic Clustering and Fibroin Gene Expression Divergence in Romanian and Imported Bombyx mori Breeds Under Standardized Rearing
Source: Insects. 2026 Jun 25;17(7):665. doi: 10.3390/insects17070665 (PMC13410149; doi:10.3390/insects17070665)
Supplement: Supplementary file 1 [file insects-17-00665-s001.zip › Supplementary_Figures_S1_S2.pdf]

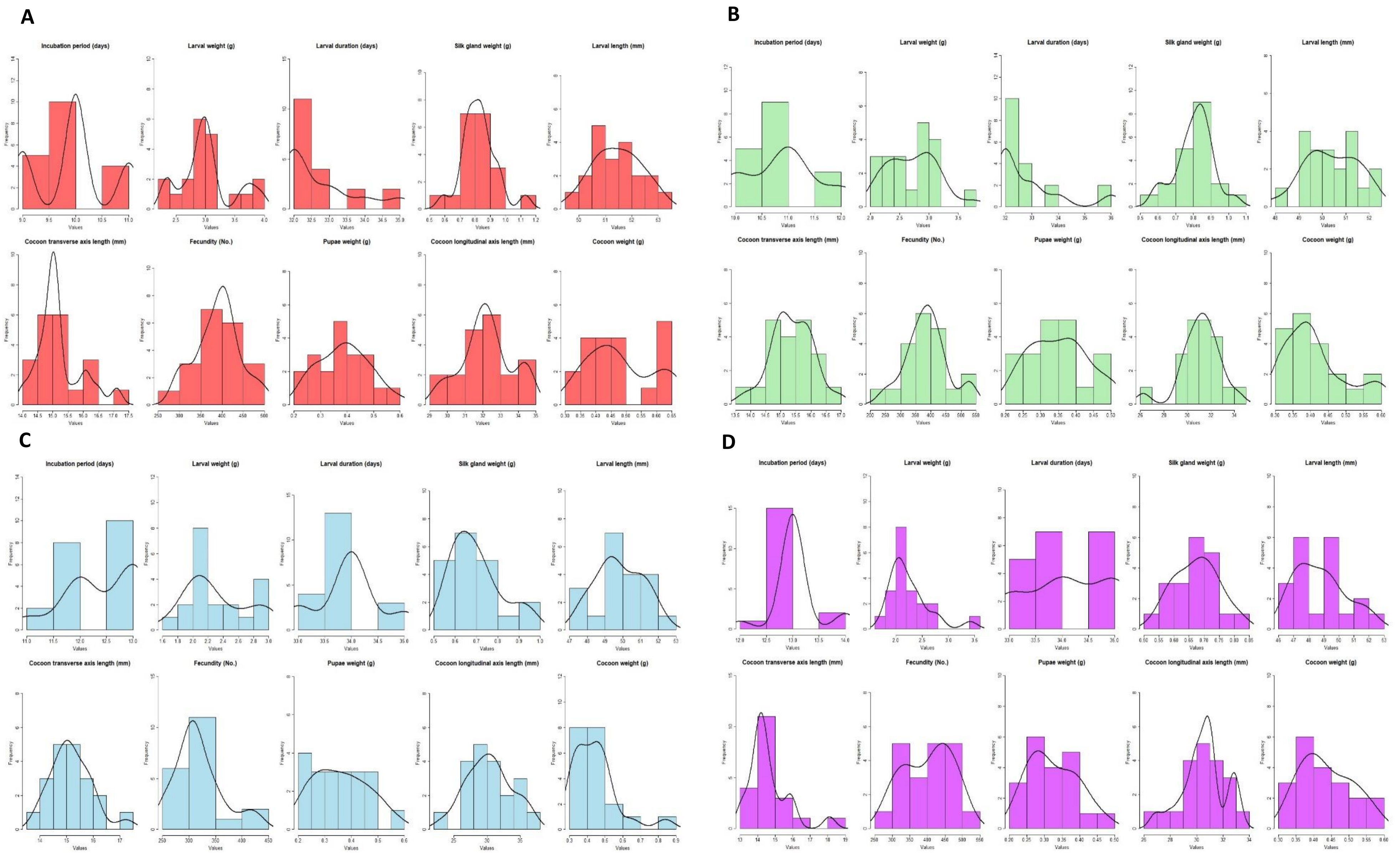

Supplementary Figure S1. Histograms of each measured biological parameters corresponding to the four local *B. mori* strains of interest: A-B1, B-JH3, C-GB, D-ACH. Histograms display the frequency of raw data using custom bins for each parameter and densities are shown as a black line for better graphical representation. All graphs were generated in RStudio.

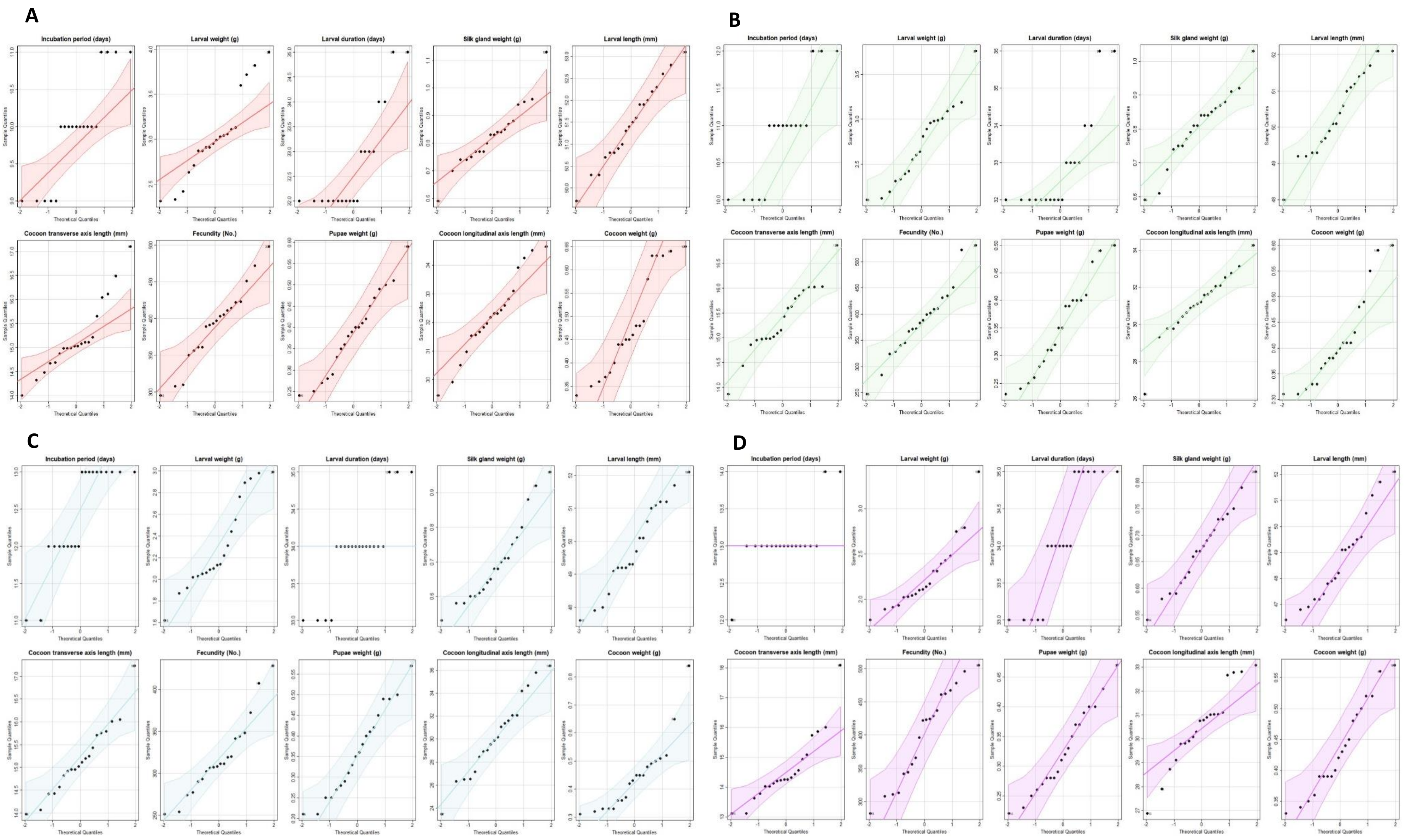

Supplementary Figure S2. Quantile-quantile (Q-Q) plots of each measured biological parameters corresponding to the four local *B. mori* strains of interest: A-B1, B-JH3, C-GB, D-ACH. Each graph displays the linear relation between quantiles drawn from the parameter distribution and the normal distribution. All graphs were generated using the *qqPlot()* function from the *EnvStats* R package in RStudio.
